# Management of a Neck Wound in a Premature Infant Using a Bilayered, Living Skin Substitute

**DOI:** 10.7759/cureus.100425

**Published:** 2025-12-30

**Authors:** Sean K Park, Nicholas De Leo, Eric J Stelnicki

**Affiliations:** 1 Plastic and Reconstructive Surgery, Cleveland Clinic Florida, Weston, USA; 2 Plastic and Reconstructive Surgery, Broward Health Medical Center, Fort Lauderdale, USA

**Keywords:** apligraf, neck wound, premature infant, skin substitute, wound healing

## Abstract

Advances in tissue engineering have enhanced our understanding of wound healing, leading to more effective management of acute and chronic wounds. Bioengineered skin constructs are increasingly favored for their minimal invasiveness and efficacy. Full-thickness neck wounds in premature neonates pose unique reconstructive challenges due to limited treatment options, high infection risk, and the potential for contracture formation. We present a rare case of an infected neck/shoulder wound in a premature infant with immunodeficiency. Following surgical debridement, a bilayered cellular skin substitute, Apligraf (Organogenesis Inc., Canton, USA) was applied to promote wound closure. Given the lack of reports describing the use of Apligraf for complex neck wounds in premature infants, this case demonstrates its potential as a viable reconstructive option in this vulnerable population, achieving early and cosmetically acceptable closure.

## Introduction

Full-thickness soft tissue wounds of the neck in premature infants represent a rare but significant reconstructive challenge. This population is uniquely vulnerable due to underdeveloped immune function, limited soft tissue reserve, impaired wound-healing capacity, and an increased risk of infection. In addition, the cervical region is both functionally and cosmetically critical. Conventional reconstructive options, such as skin grafting or flap coverage, may be contraindicated in critically ill neonates because of donor-site morbidity, hematologic instability, or prohibitive operative risk.

Advances in tissue engineering have expanded the reconstructive armamentarium for complex wounds, offering alternatives that minimize surgical trauma while promoting biologically favorable healing [1]. Bioengineered skin substitutes have been shown to enhance epithelialization, modulate inflammation, and reduce scar contracture compared with healing by secondary intention [2]. These advantages are particularly relevant in neonates, in whom even minor additional morbidity can result in disproportionately significant clinical consequences.

Apligraf (Organogenesis Inc., Canton, USA) is a bilayered, living cellular skin substitute composed of a dermal layer of neonatal fibroblasts embedded within a bovine type I collagen matrix and an overlying stratified epidermal layer of neonatal keratinocytes [3]. It is approved by the U.S. Food and Drug Administration for the treatment of venous leg ulcers and diabetic foot ulcers. Its use in other wound types, including burns and traumatic injuries, constitutes off-label application, defined as the use of an FDA-approved product for an indication not explicitly included in its approved labeling. The construct provides a physiologic extracellular matrix and delivers cytokines and growth factors that support cellular migration and wound remodeling [4].

Although bilayered skin substitutes have been reported in adult and pediatric wound management, evidence supporting their use in premature infants, particularly those with profound immunodeficiency, remains limited. In such patients, traditional grafting may be contraindicated, and prolonged healing by secondary intention carries unacceptable risks of infection and contracture. We present a rare case of a critically ill premature neonate with a complex neck wound successfully managed using a living, bilayered skin substitute, highlighting its potential role as a minimally invasive reconstructive option when standard surgical approaches are not feasible.

## Case presentation

**Figure 1:**
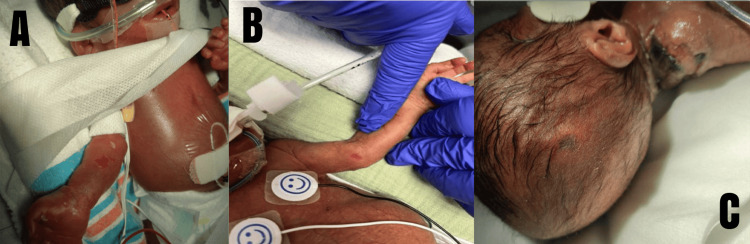
The two-day-old premature neonate developing edema in his limbs and skin tear on the right forearm (A) which was treated with topical bacitracin. During his hospital stay, he developed more skin breakdowns in other parts of the body, such as the left arm (B), and scalp (C)

A male infant was born at 29 weeks of gestation to a 26-year-old G1P0 via footling breech C-section performed for maternal pre-eclampsia. Foul-smelling amniotic fluid and nuchal cord were noted at delivery. The patient was critically ill with leukopenia, neutropenia, hypoalbuminemia, and thrombocytopenia, along with respiratory distress. The patient was placed on broad-spectrum antibiotics, including antifungals. A few days later in the neonatal intensive care unit, he was noted to have a skin tear on his right forearm, as shown in Figure 1. As the days progressed, he started to develop similar wounds in his scalp, leg, groin, and contralateral arm. The etiology of the patient’s wounds appeared multifactorial and related to his profound critical illness rather than a discrete traumatic event. These findings suggested an impaired ability to maintain skin integrity and respond to minor shear forces or ischemic insults, predisposing him to progressive tissue necrosis.

**Figure 2:**
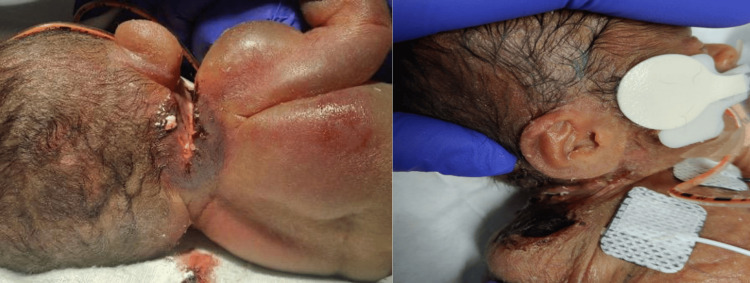
About two weeks after he was born, he started to develop a right neck wound which included significant necrosis

**Figure 3:**
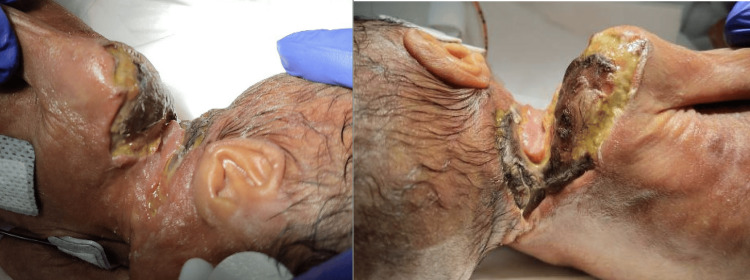
Three weeks after he was born, the right neck/shoulder wound continued to expand in size with areas of necrosis and substantial exudate

The right neck lesion was first noted on day 13 of life, with necrosis becoming apparent by day 15, as shown in Figure 2. Over the following week, daily topical antimicrobials and non-operative wound care were employed due to the patient’s hemodynamic instability and profound thrombocytopenia, which rendered early operative intervention or graft harvest unsafe. Patient’s wound continued to expand in size with areas of necrosis and exudate as shown in Figure 3.

**Figure 4:**
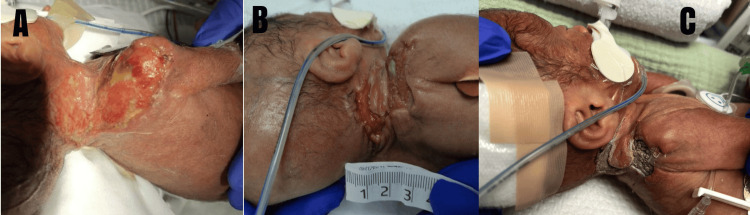
A 5x5cm right reck/shoulder wound with clean edges after the bedside debridement (A), eight days after wound debridement, wound size has shrunk down to 3x3cm with a clean, granulated tissue wound bed (B), 13 days after wound debridement, silver nitrate was applied to the wound edges (C)

On day 23, once the patient stabilized sufficiently for minor bedside procedures, debridement was performed, and culture results revealed light growth of mucor (Figure 4). Intensive antimicrobial and antifungal therapy and serial local wound care were pursued to ensure infection control prior to consideration of any reconstructive option. Silver nitrate was applied to the wound edges and daily Bactroban/Xeroform dressings were continued for one week, followed by wet-to-dry Dakin’s (0.25%) dressings. The size of the wound gradually decreased over time without any gross signs of infection (Figure 4B). Thirteen days after the debridement, the wound bed was clean, and silver nitrate was applied to promote more granulation (Figure 4C). The dressing change was transitioned to wet-to-dry normal saline. Early autologous grafting or local flap reconstruction was considered but ultimately deferred due to several contraindications: (1) the infant’s severe thrombocytopenia and anemia posed a significant risk of bleeding and graft failure, (2) ongoing fungal infection required complete resolution prior to grafting, and (3) donor-site morbidity was a major concern in a premature infant with limited available surface area. Therefore, the treatment plan prioritized infection control and wound-bed optimization before selecting a skin substitute.

**Figure 5:**
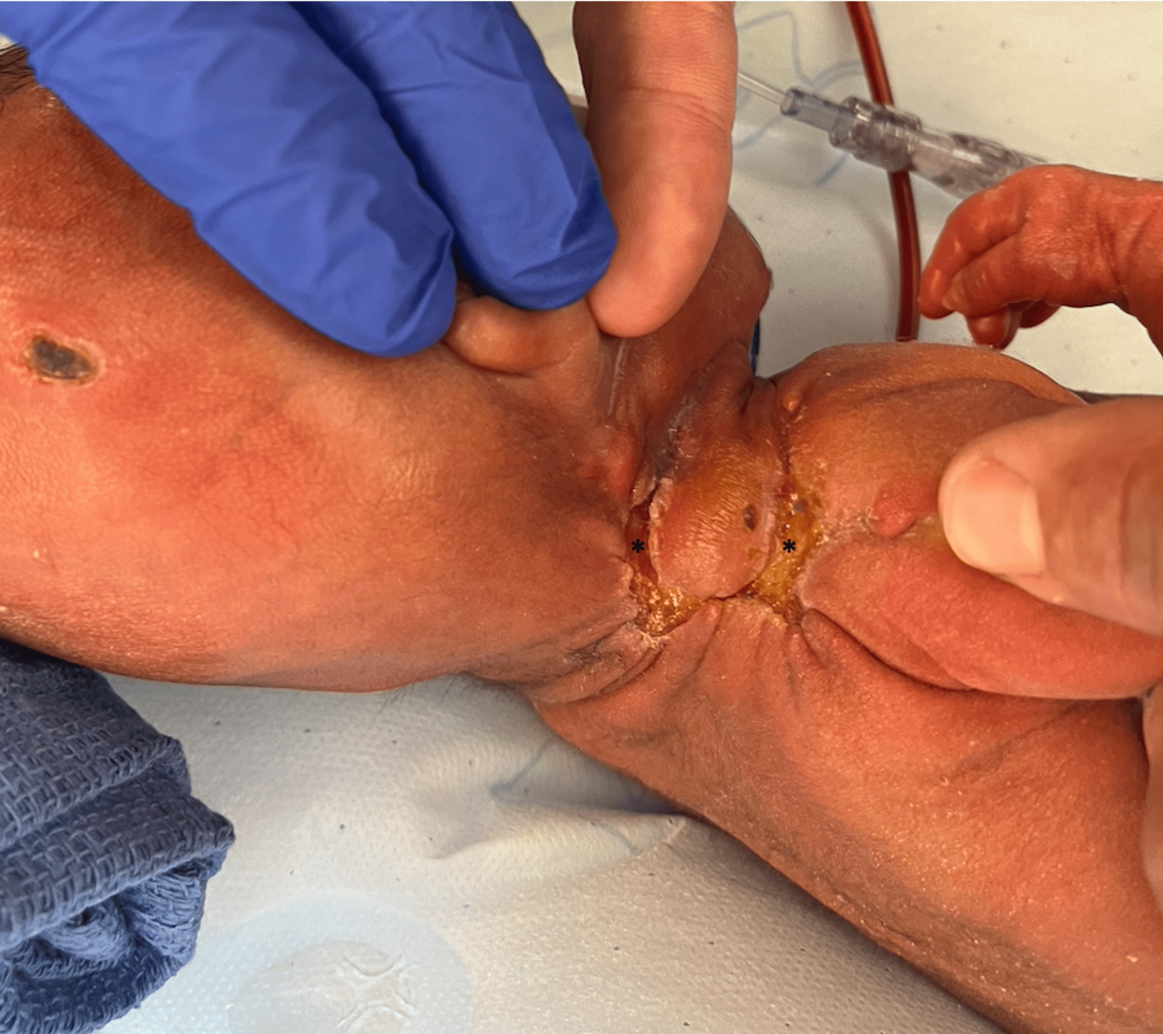
At about seven weeks of age (24 days after wound debridement), the wound continued to remain open (areas marked with ‘*’)

Despite the patient reaching seven weeks of age, the wound remained open and incompletely healed in the right neck/shoulder region (Figure 5).

**Figure 6:**
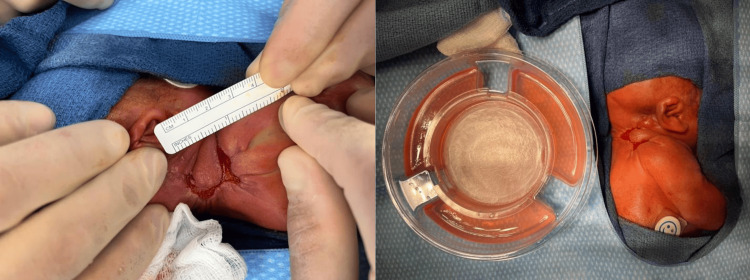
Right neck/shoulder wound bed after being curetted and irrigated. Picture on the right demonstrates a bilayered, cellular skin substitute, Apligraf used to cover the wound

**Figure 7:**
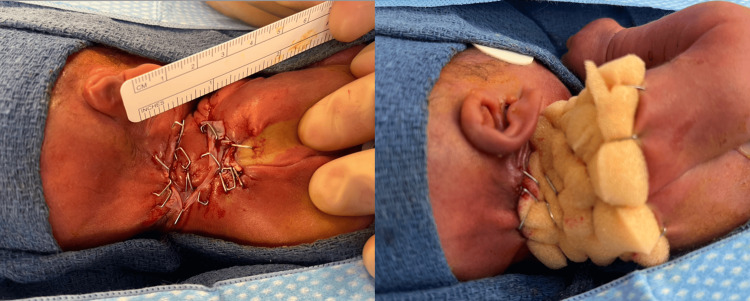
Apligraf application Once the wound was hemostatic, the allograft was removed from the Petri dish. Apligraf was cut and tailored to the size of the wound and adhered with stainless steel staples to decrease the risk of bleeding. It was then covered with an antibiotic ointment, Xeroform, and a foam bolster sponge to further stabilize the wound.

Although the wound dimensions decreased following debridement and infection control, the defect remained full-thickness with incomplete epithelialization over a mobile cervical region. Waiting longer to heal by secondary intention would pose higher risk of contracture and infection, whereas, early autografting was contraindicated due to thrombocytopenia, anemia, and limited donor sites. Apligraf application was proposed to accelerate epithelialization, once the wound demonstrated a clean, well-granulated bed with no clinical evidence of active infection. The delayed timing was intentional to ensure optimal conditions for graft take and to avoid early failure in a critically ill neonate. In the operating room, the wound was curetted and irrigated prior to applying the dermal, glossy side of Apligraf directly in contact with the wound surface (Figure 6). The skin substitute was then secured using skin staples and then covered with Bactroban, Xeroform, and a foam bolster (Figure 7).

**Figure 8:**
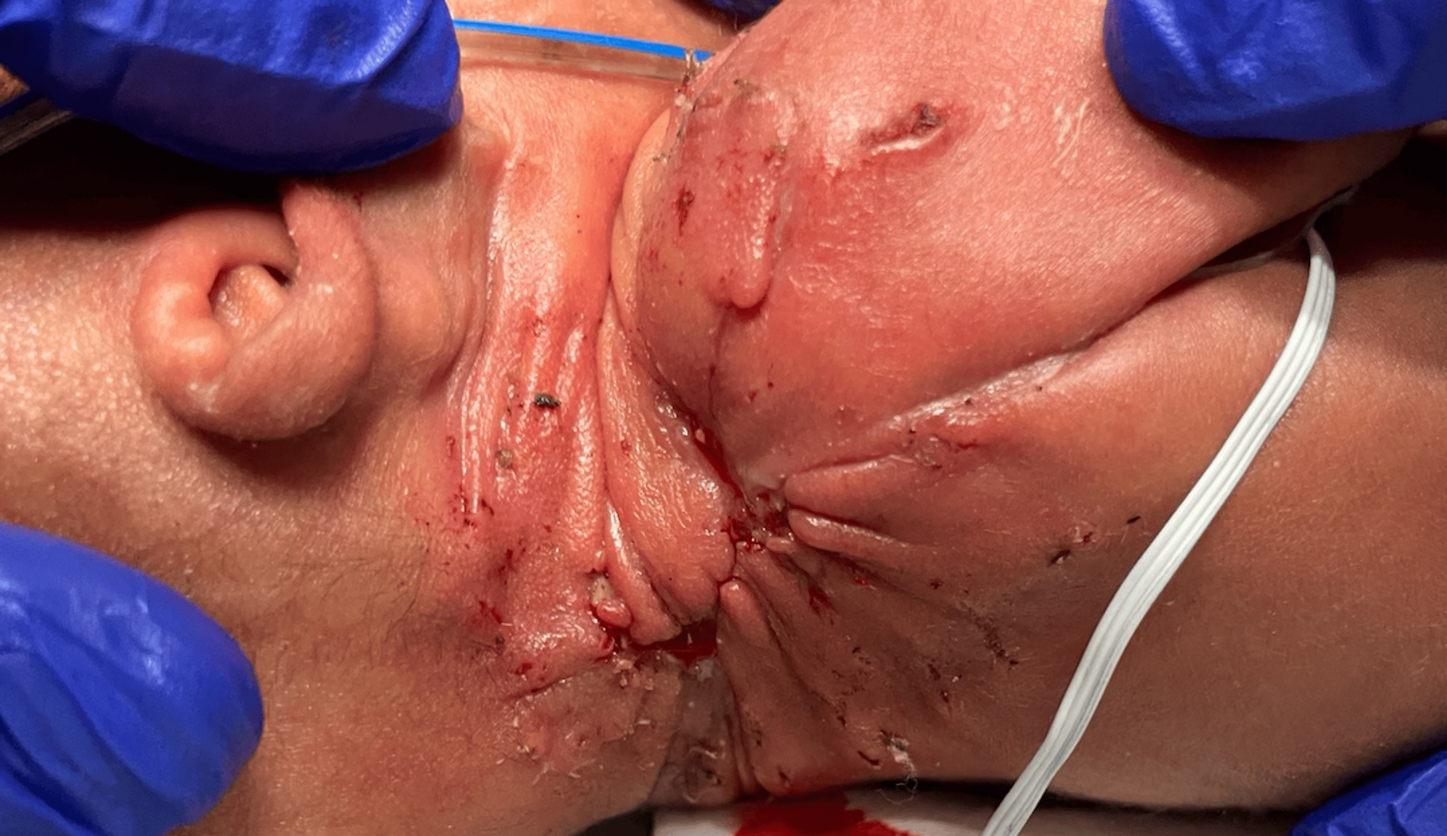
Right neck/shoulder 10 days after Apligraf application. Majority of the open wound had closed.

**Figure 9:**
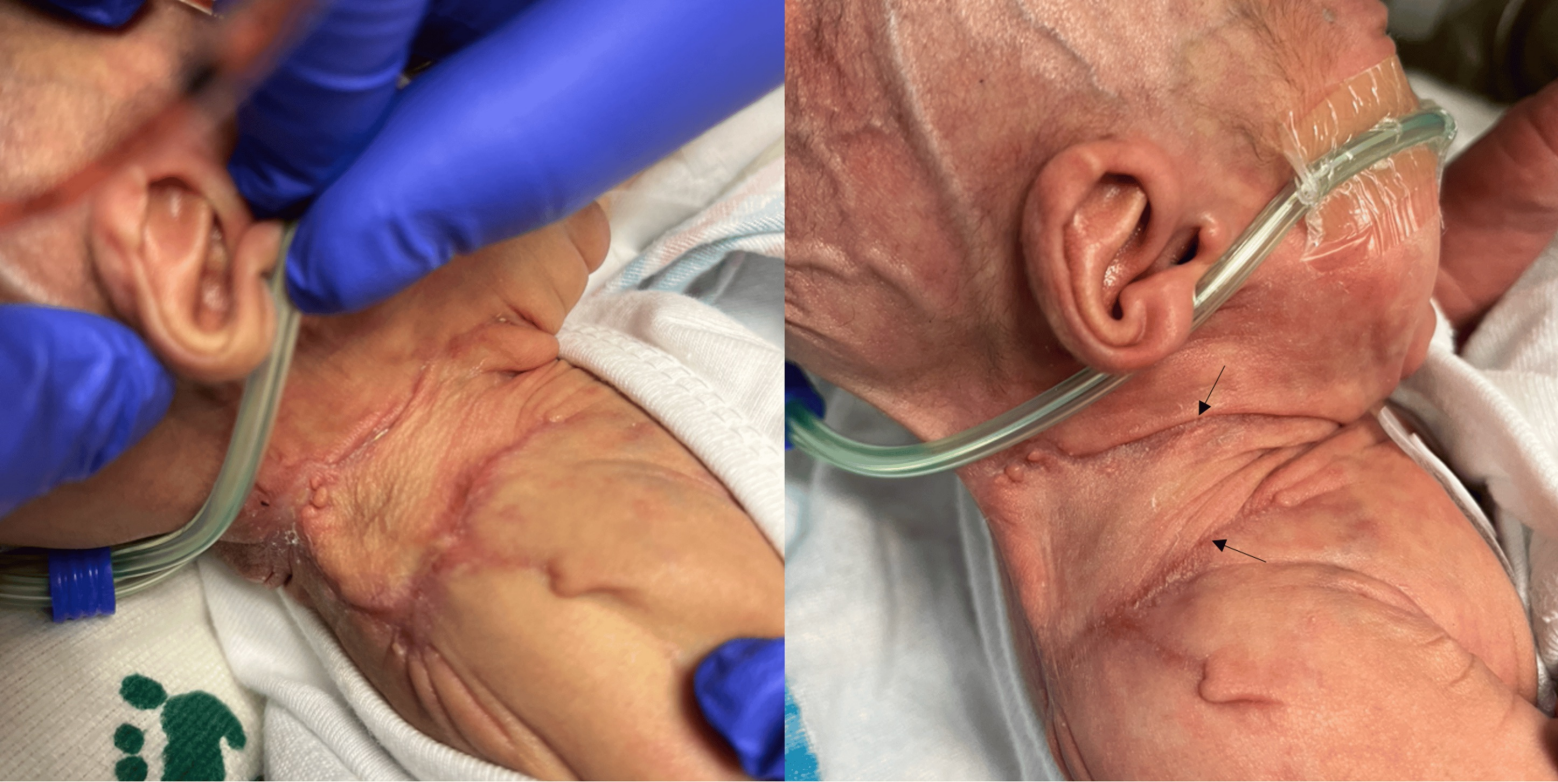
Right neck/shoulder 26 days (left) and 38 days (right) after Apligraf application. The wound remains completely closed with minimal contraction or difference in color (arrows).

Metabolic evaluation and multiple, random skin lesions raised concern for severe combined immunodeficiency (SCID); the patient was placed on neutropenic precautions. Ten days after Apligraf application, the bolster and staples were removed. An excellent allograft take was noted with a fully healed wound, as shown in Figure 8. At 26 and 38 days post-application, the wound demonstrated excellent incorporation with the surrounding skin (Figure 9). The wound remained fully epithelialized at 38 days post-application, and on bedside examination, neck mobility was preserved. However, long-term follow-up beyond this period was not available and is acknowledged as a limitation. The patient required no further surgical intervention and was later transferred for ophthalmologic evaluation of partial retinal detachment at three months of age. No external funding, sponsorship, or industry support was received for the use of this product.

## Discussion

This case underscores Apligraf as a feasible alternative to skin grafting or healing by secondary intention for full-thickness skin defects, achieving early wound closure with minimal visible contracture and color mismatch. In immunocompromised patients, timely wound closure is particularly critical to reduce the risk of infection and associated morbidity. Although the wound had decreased in diameter by the time Apligraf was applied, it remained a full-thickness defect in a high-mobility, contracture-prone region. Healing by secondary intention alone carried a substantial risk of cervical contracture. Split-thickness skin grafting was contraindicated due to the infant’s severe thrombocytopenia, anemia, and limited donor-site availability. Under these constraints, a bilayered skin substitute represented the safest and most viable option to achieve rapid epithelial coverage while avoiding the morbidity associated with operative graft harvest.

Accelerated epithelial closure within 10 days of application, uniform graft take, and early restoration of a pliable contour were temporally associated with the use of the bilayered construct. Prior to its application, the wound demonstrated stalled epithelialization despite granulation tissue formation and progressive contraction. The skin substitute provided an organized dermal matrix and viable keratinocyte layer, which appeared to facilitate epithelial bridging and mitigate further contracture. By eliminating donor-site morbidity and shortening recovery time, the bilayered construct offers meaningful clinical advantages. Eudy et al. reported similar success in a pediatric traumatic leg wound where complete healing was achieved using this construct [5]. Its extracellular matrix supports cellular ingrowth, while resident fibroblasts and keratinocytes secrete growth factors that promote wound healing. In addition, the stratum corneum component provides a physiologic barrier against mechanical injury, infection, and desiccation [3].

Alternative biologic constructs, including dehydrated amnion/chorion membrane, acellular dermal matrix, and urinary bladder matrix, have been used in pediatric wound management [2]; however, many lack a viable bilayered structure capable of providing both dermal scaffolding and immediate epidermal coverage. Comparative evidence among these products in neonates remains scarce, underscoring the relevance of this report.

Although Apligraf is FDA approved for the treatment of diabetic and venous ulcers, it is increasingly used off-label for burns, traumatic wounds, and inflammatory dermatoses, such as pyoderma gangrenosum, to accelerate wound healing [3]. Controlled studies have demonstrated faster and more cost-effective healing of chronic venous and diabetic foot ulcers compared with standard wound care, with initial costs often offset by reduced healing time and improved quality of life [4].

Given the expanding array of available skin substitutes, clinicians must understand material composition and indications to select the most appropriate construct for each patient [1]. While the outcome in this case was favorable, it represents a single experience with limited follow-up. Additionally, due to the infant’s critical condition and positioning constraints in the neonatal intensive care unit, standardized frontal and lateral photographs could not be consistently obtained. Further studies are needed to evaluate long-term durability, scar maturation, and contracture risk.

In summary, this case demonstrates accelerated wound margin reduction with minimal contracture and an acceptable cosmetic outcome. More invasive interventions, including skin grafting or flap-based reconstruction, were avoided, thereby reducing recovery time, cost, and morbidity for this high-risk patient [6,7].

## Conclusions

This case demonstrates the feasibility of using a bilayered skin substitute to achieve epithelial coverage of a full-thickness cervical wound in a premature infant with significant medical comorbidities. Although early wound contraction contributed to size reduction prior to application, the construct appeared to support timely epithelialization once the wound bed was adequately prepared. The primary value of this report lies in illustrating a minimally invasive reconstructive option for select, high-risk neonatal patients in whom conventional strategies are contraindicated. Early postoperative outcomes demonstrated acceptable incorporation and contour; however, the absence of long-term follow-up limits assessment of durability. While this report cannot establish efficacy or safety, it suggests a potential therapeutic pathway worthy of further systematic investigation.
